# Timely initiation of complementary feeding practices and associated factors among children aged 6–23 months in Dessie Zuria District, Northeast Ethiopia: a community-based cross-sectional study

**DOI:** 10.3389/fped.2023.1062251

**Published:** 2023-06-06

**Authors:** Anteneh Demelash Abate, Seid Legesse Hassen, Minwuyelet Marru Temesgen

**Affiliations:** ^1^ Amhara Public Health Institute (APHI), Health Research Development Directorate, Dessie, Ethiopia; ^2^ Amhara Public Health Institute (APHI), Health Research Development Directorate, Bahir Dar, Ethiopia

**Keywords:** complementary feeding, Dessie Zuria, Amhara region, Ethiopia, timely initiation

## Abstract

**Introduction:**

Ethiopia has one of the highest infant and child mortality rates in the world. Starting from the age of 6 months, breast milk alone is not sufficient to cover all nutritional requirements. Infants and young children are at an increased risk of undernutrition. Complementary feeding must, therefore, begin at the age of 6 months. Infant and young child nutrition is a critical factor in human health, nutrition, survival, growth, and development. Therefore, the aim of this study is to evaluate the timely initiation of complementary feeding practices and associated factors in children aged 6–23 months in the Dessie Zuria District of North Ethiopia.

**Methods:**

A community-based cross-sectional study design was used for the period between 16 March and 30 March 2019. The study included 770 mother–child pairs aged 6–23 months. A multistage sampling method was used to choose the study participants. Using a simple random sampling technique, nine kebeles in the district were selected from a total of 31, and from 103 Gotts or villages, 31 were selected with 770 HHs out of 2,329 HHs with children aged 6–23 months. Data were collected using a pretested semistructured interviewer-administered questionnaire, which was then entered into Epi Data version 3.1 statistical software before being transferred to SPSS version 21 for further analysis. To summarize the data, descriptive statistics were used, which included a simple frequency table and figures. To evaluate factors, bivariate and multivariable logistic regression were used. A *p*-value of less than 0.05 was used to determine statistical significance.

**Results:**

The percentage of children who started complementary feeding practices on time was 70.9. Maternal occupation [AOR = 5.51, 95% CI (1.61–18.81)], radio availability [AOR = 2.03, 95% CI (1.32–3.12)], antenatal care follow-up [AOR = 6.19, 95% CI (4.08–9.40)], place of delivery [AOR = 5.06%, CI (3.34–7.68)], and postnatal care follow-up [AOR = 4.32, 95% CI (2.77–6.72)] were found to be the factors for the timely initiation of complementary feeding.

**Conclusion:**

When compared with WHO cutoff points, timely initiation of complementary feeding practice was relatively low in the study area. Maternal occupation, radio availability, ANC follow-up, place of delivery, and postnatal care visit were all significantly associated with the timely initiation of complementary feeding.

## Introduction

Complementary feeding is defined as the process of beginning complementary food when breast milk alone is insufficient to meet an infant's nutritional needs, and as a result, other foods and fluids are required. At 6 months of age, infants should receive safe and nutritionally adequate complementary foods, according to global public health recommendations (1, 2). Appropriate complementary feeding encourages growth and prevents stunting in children aged 6–23 months. Improving infant and toddler feeding is critical to ensuring optimal health, nutrition, and development (3–5). Undernutrition is still one of the most serious public health issues that affect people of all ages throughout their lives. The most vulnerable victims are typically infants and children. Undernutrition has been the leading single cause of infant and child mortality worldwide, with 3.4 million children dying every year from malnutrition-related causes associated with inappropriate complementary feeding during the first year of life (6, 7).

According to the report on the situation of IYCF in Venezuela, inappropriate complementary feeding practices continue to be the leading cause of undernutrition in children under the age of 2 (8). Undernutrition was responsible for 50%–70% of the burden of diarrheal diseases, measles, malaria, and lower respiratory infections, according to the global consultation report (9).

One of the major platforms for program implementation in Ethiopia to shape Infant and Young Child Feeding (IYCF) demand and practice is through existing community-based nutrition programs. The strategy is to support and empower the government's Community-Based Health Extension Program and Health Development Army to mobilize their communities, deliver important preventive messages, and offer counseling to promote optimal IYCF behaviors (10, 11).

Undernutrition remains a major public health issue, with the consequences of undernutrition during the first 1,000 days being largely irreversible if not addressed appropriately. The main causes of undernutrition are a lack of appropriate breastfeeding and complementary feeding practices (12, 13). Globally, undernutrition is directly or indirectly responsible for at least 35% of deaths in children under the age of 5. In developing countries, an estimated 32% and 10% of children under the age of 5 are stunted and wasted, respectively, and nearly 30% of infants, children, adolescents, adults, and older people are currently suffering from one or more of the multiple forms of malnutrition, with undernutrition accounting for 49% of deaths among under-5 children every year (14, 15).

According to the Ethiopian Demographic Health Survey (EDHS) 2016, 38% of children under the age of 5 are stunted, and stunting is more prevalent in rural areas (40%) than in urban areas (25%). There are also some regional differences; stunting rates range from 46% in the Amhara region to 15% in Addis Ababa (16). According to a comparison of Infant and Young Child Feeding (IYCF) indicators in Amhara and nationally, conducted by Alive and Thrive, the rates of timely introduction of complementary feeding were only 35% in Amhara and 46% nationally (17). According to the recommendation of the World Health Organization (WHO)/United Nations International Children's Fund, the prevalence of timely initiation of complementary feeding practices was low in different parts of the countries studied. In several parts of the countries surveyed, the prevalence of timely initiation of complementary feeding practices was low, 60.5%, 52.8%, 40%, and 63% in Hiwot Fana Specialized Hospital, Axum Town, Kamba District, South West Ethiopia, and Lalibela District, respectively (18, 19). Implementing proper IYCF practices, including exclusive breastfeeding from birth to 6 months and appropriate complementary feeding for children aged 6–23 months, is critical for preventing stunting and nutrition-related morbidity and mortality (20–22). Because breast milk alone is not sufficient to maintain a child's optimal growth, the United Nations International Children's Fund (UNICEF) and WHO recommend introducing solid food to infants at the age of 6 months. Undernutrition becomes more prevalent in many countries during the transition period (ages 6–23 months) due to an increase in infections and poor feeding practices (22, 23). A study, conducted in Bishoftu, Oromia, Ethiopia, found that the main reasons for not initiating complementary feeding practices on time were mothers’ unemployment, no place for delivery, a lack of knowledge of the recommended duration of exclusive breastfeeding, and not spending time to initiate breastfeeding following delivery (24). Despite the implementation of IYCF practices through HEWs in the Dessie Zuria District, the prevalence of timely initiation of complementary feeding practices and their associated factors is unknown. Thus, the findings of our study would be essential to provide evidence for decision-makers, health facilities, and care providers to take measures to target those children at the highest risk of undernutrition caused by malpractices. In addition, the study findings are important, as they can provide scientific evidence that is required to solve problems related to under- and malnutrition and would help initiate a plan. As a result, the purpose of this study is to determine the prevalence and associated factors of the timely initiation of complementary feeding practices in the Dessie Zuria District.

## Materials and methods

### Study design

The research was conducted between 16 March and 30 March 2019, in the Dessie Zuria district. A community-based cross-sectional study design was used. Dessie Zuria District is located in the South Wollo Zone of Ethiopia's Amhara national region state and is 401 km from the Ethiopian capital city. Dessie City serves as the administrative center for this district; other small towns in the Dessie Zuria District include Guguftu, Ayata, Geisha, and Mote. According to the data of Amhara National Regional State, the district is the fourth most populous of the 23 districts in the South Wollo Zone. It is administratively divided into one urban and 30 rural kebeles (the smallest administrative unit), with a total population of approximately 176,836 people. A district-based census was conducted by the Bureau of Finance and Economic Development (BOFED) in 2018. There were 90,933 males (51.4%) and 85,903 females (48.6%), and 4.39% were children aged 6–23 months. The district has a total population of 41,125 households. At the time, the community was served by eight health centers, one urban health post, and 30 rural health posts. Agriculture is the primary occupation of the inhabitants, with only a small proportion of the population engaged in trading. The district is well known for its poor infrastructure and limited social services. The district's major crops are both rain-fed and irrigated crops, such as maize, millet, teff, barley, wheat, and vegetables (25).

The study's source populations were all mothers with at least one child aged 6–23 months who had lived in the study area for more than 6 months, and the mothers were chosen at random. The sample size was calculated using Epi-info version 7, with the following assumptions in mind: the proportion of timely initiation of complementary feeding in the Amhara Region (35%) in EDHS 2011 (26), 95% confidence interval (CI), 5% margin of error, and 10% non-responsive rate. The final sample size was 770 when two design effects were used. According to data obtained from the District Health Office, the district has 31 kebeles (one urban and 30 rural), with 7,764 households having children aged 6–23 months living in the district. A multistage sampling method was used to choose the study participants. A simple random sampling technique was used to choose nine kebeles in the district out of a total of 31; and 31 Gotts or villages (30%) were chosen at random among 103 Gotts or villages, with 770 HHs from the total of 2,329 HHs with children aged 6–23 months.

**Figure 1:**
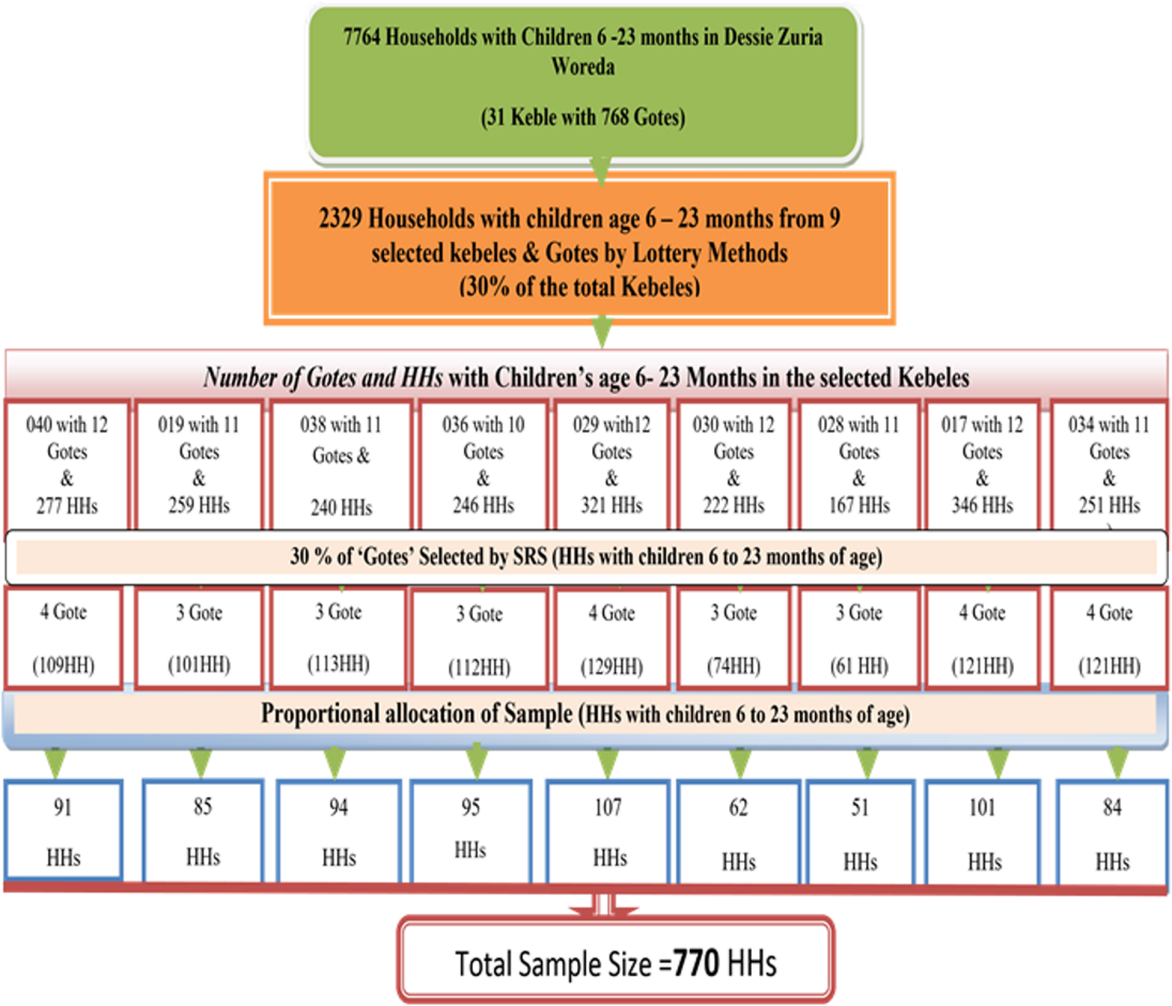
Diagram of the sampling procedure in the Dessie Zuria District, South Wollo Zone, and the Amhara Region. March 2019.

Based on the number of HHs with children aged 6–23 months, 770 HHs with children aged 6–23 months were proportionally assigned to each Gott. The sampling frame was a list of households from selected kebeles with children aged 6–23 months obtained from the most recent routine monthly nutritional screening registration list prepared for implementing the community health information system (CHIS) in each selected health post/kebele by health extension workers before the actual data collection date. The total number of HHs in each Gott study unit was chosen at random using lottery methods. If there was more than one child aged 6–23 months in the same household, only one index child was chosen at random to collect information (Figure 1).

### Outcome measures

The outcome of this study was the timely initiation of complementary feeding. Complementary feeding is defined as the point at which food intake other than breast milk begins between 6 and 8 months of age. Starting 6 months earlier or 8 months later is not considered timely. Under 6 months and over 8 months, complementary feeding initiation was recorded as “0” (No/not on time); complementary feeding initiation at 6–8 months was recorded as “1” (Yes/is on time).

### Covariates/exposure variables

Independent variables were sociodemographic characteristics, source of information, individual factors (awareness levels of mothers, husband support, and the influence of others), mothers’ health service utilization history, and mothers’ obstetric history.

### Data collection and quality assurance

Data were collected by home visits from mothers (caregivers) with children aged 6–23 months. The questionnaire was adapted from different studies in the literature and indicators to assess IYCF practice in a research setting. The questionnaire was pretested on 5% of the sample of mothers with children aged 6–23 months at “Tebasit Kebele,” one of the Kebeles in Dessie Zuria District that did not include the main “Kebeles” but had similar settings within the study areas. Data collectors were trained so that they all had a common understanding of the study's objectives and all of the questions in the questionnaire. Data collection was monitored and the principal investigator checked each questionnaire daily for completeness. We believe that the internal consistency estimate is within an acceptable range. After all data were collected and checked for accuracy, they were edited, coded, and entered into Epi Data version 3.1 before being exported to the SPSS software version 21 package for analysis.

### Statistical analyses

Descriptive statistics were used to determine the distribution of the study participants, which included frequency distribution, cross-tabulation, and summary measures. We used the Hosmer–Lemeshow test for determining the model fitness to conduct binary logistic regression analyses to identify the factors associated with the timely initiation of complementary feeding practices. A statistically significant association was defined as one with a *p*-value of less than 0.05. This can be used by individually entering all independent variables into the bivariate analysis model and applying these variables with a *p*-value of 0.25 to multiple regression models. Finally, we employed a backward stepwise regression technique, to assess the strength of the association between the dependent and the independent variables. Adjusted odds ratios (AORs) with a 95% confidence interval were calculated. Finally, the study's findings were presented as statements, text, tables, and charts.

### Operational and standard definition

• Timely introduction of complementary feeding: The proportion of infants who received solid, semisolid, and soft foods in addition to breastmilk or a breastmilk substitute at 6–8 months of age.
• Information source: the provision of information in various forms such as radio, television, health extension workers, health workers, and the Health Development Army.
• Untimely initiation of complementary feeding: the initiation of complementary feeding for an infant and young child before and after the age of 6 months.
• Knowledge assessment: twelve questions were used to assess the awareness levels of mothers. Mothers with mean values greater than the mean were considered to have good awareness, whereas respondents with mean values less than the mean were considered uninformed.

## Results

### Socio-demographic characteristics of the study participants

This study included 770 mothers with children aged 6–23 months, resulting in a 100% response rate. The mean age of the mothers was 28.25 years with a standard deviation of 5.654, and the mean age of the children was 14.35 months with a standard deviation of 4.479. One-third (39.2%) of the children were between the ages of 12 and 17 months. As for the religious background of the mothers, 722 (93.8%) were Muslim, 47 (6.1%) were Orthodox Christians, and only 1 (0.1%) was a Protestant. A total of 611 (79.4%) of all respondents were married mothers. The participants were exclusively Amhara ethnic groups. Nearly half of the children, totaling 409 (53.1%), were male, while the remaining 361 (46.9%) were female. In terms of maternal educational status, 242 (31.4%) of the participants could not read or write, while 22 (2.9%) attended college or higher education. Six hundred and sixty-six (86.5%) of the mothers lived in rural areas. In terms of the availability of information material in the home, only 36 (4.7%) and 255 (33.1%) of respondents had television and radio, respectively. In terms of the respondents’ occupational status, 453 (58.8%) were housewives. Based on the average monthly income, only 137 (17.8%) of the respondents earned more than 500 Ethiopian birrs (Table 1).

**Table 1:** Socio-demographic characteristics of mothers and children aged 6–23 months in the Dessie Zuria District, Ethiopia, March 2019.

Variables	Response category	Frequency	%
Age of the child in a month	6–11 Months 12–17 Months	234	30.4
	18–24 Months	302	39.2
	6–11 Months 12–17 Months	234	30.4
Age of the mother in years	<20 Years	63	8.2
	20–24 Years	137	17.8
	25–29 Years	278	36.1
	30–34 Years	175	22.7
	>35 Years	117	15.2
Place of residence	Urban	104	13.5
	Rural	666	86.5
Marital status	Married	611	79.4
	Single	63	8.2
	Divorced	67	8.7
	Widowed	29	3.8
Maternal education status	Cannot read and write	242	31.4
	Can read and write only	206	26.8
	Primary education (1–8)	238	30.9
	Secondary education (9–12)	62	8.1
	College diploma and above	22	2.9
Maternal occupation	Housewife	453	58.8
	Farmer	199	25.8
	Merchant	61	7.9
	Government worker	37	4.8
	Daily laborer	20	2.6
Father education status	Cannot read and write	236	30.6
	Can read and write only	222	28.8
	Primary education (1–8)	227	29.5
	Secondary education (9–12)	51	6.6
	College diploma and above	34	4.4
Average monthly income in birr	<500	633	82.2
	>500	137	17.8

### Maternal reproductive history and health service utilization

The study included 743 (96.5%) mothers with two or fewer deliveries and 296 (38.4%) mothers with more than one child and a delivery interval of 3 or more years. A total of 455 (59.1%) of all respondents had four or more families living in their home. A total of 399 (51.8%) of all respondents gave birth in a health facility. With regard to the ANC follow-up, 393 (51%) of the respondents received ANC services, and 171 (43.5%) of these women received ANC four times during their previous pregnancy. With regard to PNC follow-up, 305 (39.6%) of the respondents availed PNC services, and 99 (32.4%) of these mothers had three or more follow-up visits. After the sixth month of delivery, 511 (66.4%) of the respondents had home visits from healthcare professionals. Almost all 764 infants (99.2%) were immunized, and 678 (88.1%) were certified as having completed immunization (Table 2).

**Table 2:** Maternal obstetric history and utilization of health services in the Dessie Zuria District, Ethiopia, March 2019.

Variables	Response category	Frequency	%
Family size in the house	2–3 Family	209	27.1
	4–5 Family	455	59.1
	≥6 Family	106	13.8
Number of deliveries/parity	1–2 Child	743	96.5
	3 and above Child	27	3.5
Less than 5 children	1–2 child	726	94.3
	3 and above	44	5.7
Place of delivery	Home	399	51.8
	Health facility	371	48.2
ANC visits	Yes	393	51
	No	377	49
Frequency of ANC visits	One time	115	29.3
	Two times	27	6.9
	Three times	80	20.4
	Four times	171	43.5
PNC visits	Yes	305	39.6
	No	465	60.4
Frequency of PNC visits	One time	101	33.1
	Two times	105	34.4
	Three and above	99	32.5
Vaccination	Yes	764	99.2
	No	6	0.8
Health professional visits	Yes	511	66.4
	No	259	33.6

### Individual factors associated with proper complementary feeding initiation

Of the 770 participants, 591 (76.8%) were aware about complementary feeding initiation, while the remaining 179 (23.2%) of the respondents had poor awareness because they correctly scored below the mean. A total of 491 (63.8%) of mothers who started complementary feeding on time had husband support. Six hundred and thirty-five (82.5%) of these mothers had heard an educational message about the importance of starting complementary feeding on time (Table 3).

**Table 3:** This table shows mothers’ awareness levels of timely initiation of complementary feeding in the Dessie Zuria District of Ethiopia in March 2019.

Variables	Response category	Frequency	%
Importance of complementary food	Important for the growth and health of the child	655	85.1
	Exposed the child to illness	108	14
	I do not know	7	0.9
Age to start complementary feeding	Before 6 months	141	18.3
	At 6 months	627	81.4
	After 6 months	2	0.3
Types of food the child should be started	Liquid	88	11.4
	Semisolid	682	88.6
	Solid	88	11.4
Frequency of feeding per a day	One to three times	434	56.4
	Three to five times	336	43.6
Information about the time to start	Yes	635	82.5
	No	135	17.5
Having awareness	Yes	591	76.8
	No	179	23.2

**Figure 2:**
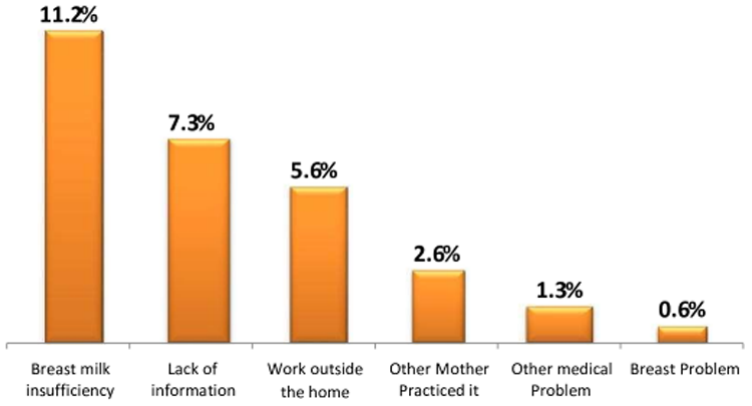
Reasons for early complementary feeding initiation in the Dessie Zuria District, Ethiopia, March 2019.

**Figure 3:**
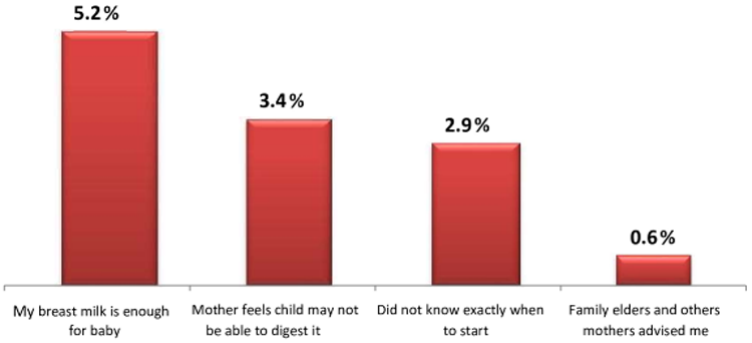
Reasons for complementary feeding delay in the Dessie Zuria District, Ethiopia, March 2019.

Mothers who did not introduce complementary feeding on time did so due to a lack of breast milk 86 (11.2%) and 40 (5.2%) sufficient of breast milk for early and late commencement of complementary feeding, respectively, of 224 mothers) (Figures 2, 3).

### Source of information on the appropriate initiation of complementary feeding

Health extension workers were the source of information for 581 (75.5%) of the mothers, and 627 (81.4%) respondents were aware of the optimal time to begin supplemental feeding methods (Table 4).

**Table 4:** Information sources on complementary feeding in the Dessie Zaria District, Ethiopia, March 2019.

Variables	Response category	Frequency	%
Health professionals	Yes	518	67.3
	No	252	32.7
Health extension workers	Yes	581	75.5
	No	189	24.5
From other mothers	Yes	91	11.8
	No	679	88.2
From the health development army	Yes	127	16.5
	No	643	83.5
Radio or/and TV	Yes	259	33.6
	No	511	66.4

### Timely initiation of complementary feeding practices

**Figure 4:**
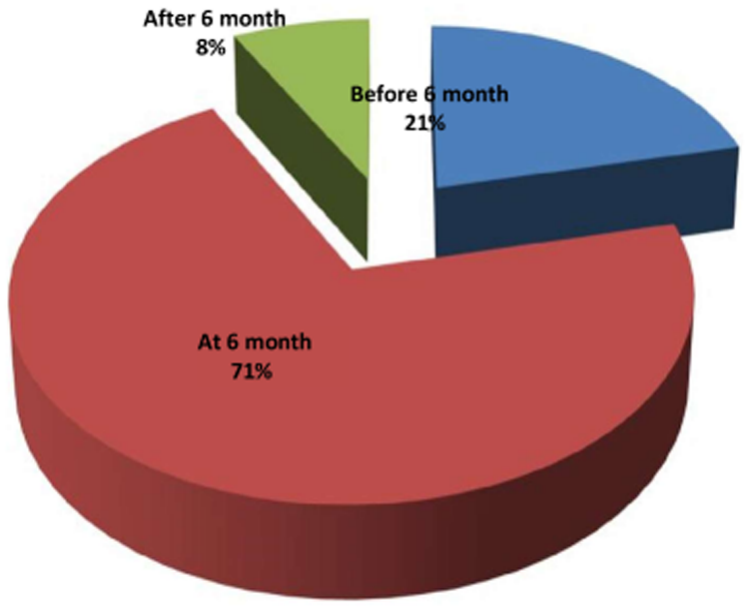
Practice of timely complementary feeding initiation in the Dessie Zuria District, Ethiopia, March 2019.

Approximately 71% of parents initiated complementary feeding on time for their children. Six hundred and sixty (67%) of children were started with semisolid food, and more than half (54.4%) of them were fed three to five times per day (Figure 4, Table 5).

**Table 5:** Information sources on complementary feeding in the Dessie Zaria District, Ethiopia, March 2019.

Variables	Response category	Frequency	%
Started CF at 6 months	No	224	29
	Yes	546	71
Having husband support	Yes	490	63.6
	No	56	7.3
Type of food started during CF	Semisolid (porridge)	516	67
	Liquid	280	36.4
	Family-type food	103	13.4
	Packed and bottled food	3	0.4

### Factors associated with the timely initiation of complementary feeding

Bivariate and multivariate analyses were used to investigate the parameters associated with the timely initiation of complementary feeding practices. In the bivariate logistic regression analysis, factors such as the place of residence, maternal occupation, radio availability, place of delivery, ANC follow-up, and PNC follow-up were all associated with the timely initiation of complementary feeding and were imported into the multivariate logistic regression analysis. However, backward logistic regression analysis revealed that maternal occupation, radio availability, place of delivery, ANC, and PNC follow-up were significantly associated with the timely initiation of complementary feeding.

As a result, housewife mothers were 5.51 times more likely to practice the timely initiation of complementary feeding than daily laborers [AOR = 5.51, 95% CI (1.61–18.81)]. Households with a radio were 2.03 times more likely than households without a radio to practice the timely initiation of complementary feeding [AOR = 2.03, 95% CI (1.32–3.12)]. Mothers who received ANC follow-up visits were 6.19 times more likely than mothers who did not to initiate complementary feeding on time for children aged 6–23 months [AOR = 6.19, 95% CI (4.08–9.40)]. Similarly, mothers who gave birth in a health facility were 5.06 times more likely than those who gave birth at home to initiate complementary feeding on time [AOR = 5.06, 95% CI (3.34–7.68)]. Mothers who received PNC follow-up were also 4.32 times more likely than those who did not have follow-up to initiate complementary feeding on time [AOR = 95% CI (2.77–6.72)] (Table 6).

**Table 6:** Depicts the association between some selected characteristics and the timely beginning of supplementary feeding in Ethiopia's Dessie Zuria District in March 2019 (*n* = 770).

Variables	Response	Timely initiation of complementary feeding			COR (95% CI)	AOR (95% CI)	*p*-value
		Yes *n* (%)	No *n* (%)	Total *n* (%)			
Place of residence	Urban	62 (59.6%)	42 (40.4%)	104 (100%)	.55 (.36–.85)		
	Rural	484 (72.7%)	182 (27.3%)	666 (100%)	1		
Maternal occupation	Housewife	347 (76.6%)	106 (23.4%)	453 (100%)	4.91 (1.95–12.33)	5.51 (1.61–18.81)*	0.006
	Farmer	131 (65.8%)	68 (34.2%)	199 (100%)	2.89 (1.12–7.40)		
	Merchant	38 (62.3%)	23 (37.7%)	61 (100%)			
	Govt worker	22 (59.5%)	15 (4.5%)	37 (100%)			
	Daily laborer	8 (40%)	12 (60%)	20 (100%)	1		
Availability of radio	Yes	201 (78.8%)	54 (21.2%)	255 (100%)	1.83 (1.29–2.60)	2.03 (1.32–3.12)**	0.001
	No	345 (67%)	170 (33%)	515 (100%)	1		
Average monthly income	<12.5 $	441 (69.7%)	192 (30.3%)	633 (100%)	1		
	≥12.5$	105 (76.6)	32 (23.4%)	137 (100%)	1.42 (.92–2.19)		
Place of delivery	Home	220 (55.1%)	179 (44.9%)	399 (100%)	1		
	Health facility	326 (87.9%)	45 (12.1%)	371 (100%)	5.89 (4.07–8.52)	5.06 (3.34–7.68)**	<0.001
ANC visit	Yes	349 (88.8%)	44 (11.2%)	393 (100%)	7.24 (4.99–10.52)	6.19 (4.08–9.40)**	<0.001
	No	197 (52.3%)	180 (47.7%)	377 (100%)	1		
PNC visit	Yes	262 (85.9%)	43 (14.1%)	305 (100%)	3.88 (2.67–5.63)	4.32 (2.77–6.72)**	<0.001
	No	284 (61.1%)	181 (38.9%)	465 (100%)	1		

* Maternal occupation (*p*-value < 0.05).

** Availability of radio, place of delivery, ANC visit, and PNC visit (*p*-value ≤ 0.01).

## Discussion

### Main findings

According to this study, 71% of children aged 6–23 months started complementary feeding at 6 months. However, this percentage is low when compared with the WHO cutoff point of greater than or equal to 80% for the timely initiation of supplementary feeding (27). This disparity can be attributed to low maternal literacy and the utilization of maternal health services. The rates of this study's findings are lower than those of previous studies in Bangladesh (83.1%), Nepal (77%), and Abyei Adi, Ethiopia (79.7%) (7, 28, 29). Poor household socioeconomic level, lower maternal literacy, poor health-seeking behavior, relatively low utilization of maternal healthcare facilities, and methodological variation between the study and the nature of the study population could all be the reasons for the low rates.

However, the rates of this study’s findings are greater than the 51% reported in the 2011 Ethiopian Demographic Health Survey (EDHS), 52.8% in Axum, 63% in Lalibela, 56.5% in Lasta, 56.4% in Enemay, 55% in Addis Ababa, and 60.5% in Harar Town's Hiwot Fana Hospital (7, 18, 19, 22). This could be attributed to the relatively high utilization of health services such as antenatal care, institutional delivery, and postnatal care, as well as the efforts of health extension workers. For the past 2 years, health extension workers have made house-to-house visits to assist families in gaining access to basic health services and to provide home-based health education, including the promotion of appropriate IYCF practices and the gaining of support of organizations worldwide in order to improve IYCF practices in the study area and to study the time difference. So, in comparison with other studies, in this study, better awareness has been found in mothers of the correct time for starting complementary feeding and of such feeding practices, and these could be the reasons for the gap that exists between this study and other studies.

The results are consistent with those obtained in the blocks of Doiwala in Dehradun District in India (70.1%) and Arsi, Ethiopia (72.5%) (30). Maternal occupation and radio availability were two of the potentially associated factors investigated with regard to the timely initiation of complementary feeding practices of children aged 6–23 months in the Dessie Zuria District. The place of delivery, ANC, and PNC follow-up were found to be significantly related to the timely implementation of complementary feeding practices of children aged 6–23 months.

This study found that housewife mothers were 5.51 times more likely than daily laborer mothers to initiate complementary feeding practices on time for children aged 6–23 months [AOR = 5.51, 95% CI (1.61–18.81)]. This study finding is consistent with that conducted in Mekele, northern Ethiopia, where housewives were significantly associated with the timely initiation of complementary feeding practices of children aged 6–23 months [AOR = 2.34, 95% CI (1.40–3)] (31, 32). This could be attributed to housewives typically staying at home with their children and having enough time for frequent breastfeeding at 6 months and then to begin complementary feeding on time.

This study also found that households with a radio were 2.03 times more likely to initiate complementary feeding practices on time than households without a radio [AOR = 2.03, 95% CI (1.32–3.12)]. This finding is supported by research from Lasta, which found that mothers exposed to public media had a significantly higher likelihood of initiating complementary feeding practices on time [AOR = 2.59, 95% CI] (33). This could be attributed to nutrition information being broadcasted on multiple radio channels.

This study also found that mothers who received ANC follow-up visits were 6.19 times more likely to initiate complementary feeding on time than mothers who did not [AOR = 6.19, 95% CI (4.08–9.40)]. This finding is similar to that of a study conducted in Addis Ababa, Ethiopia, where mothers who received ANC were significantly associated with the timely initiation of complementary feeding of children aged 6–23 months [AOR = 4.78, 95% CI (1.30–17.47)] (34). One possible reason is that visiting a healthcare facility during pregnancy provides an excellent opportunity to obtain health information and counseling on complementary feeding.

According to the findings of this study, mothers who gave birth in a health facility were approximately 5.06 times more likely than those who gave birth at home to initiate complementary feeding on time of children aged 6–23 months [AOR = 5.06, 95% CI (3.34–7.68)]. This finding is consistent with the Enemay District study, which found that mothers who gave birth in a health facility were significantly associated with the timely initiation of complementary feeding practices of children aged 6–23 months [AOR = 2.39, 95% CI (1.40–4.06)] (35). This could be attributed to the fact that mothers who gave birth in health institutions would receive advice and counseling from health professionals regarding breastfeeding and child feeding practices, whereas home-delivered mothers would lack adequate information on recommended infant feeding practices and would be most affected by the inadequate infant feeding practices of their communities.

According to the study's findings, mothers who received PNC follow-up visits were 4.32 times more likely to initiate complementary feeding on time of children aged 6–23 months than mothers who did not [AOR = 4.32, 95% CI (2.77–6.72)]. This finding is similar to that of a study carried out in Abyei-Adi, Ethiopia, where mothers who had PNC follow-up visits were significantly associated with the timely initiation of complementary feeding of children aged 6–23 months [AOR = 2.80, 95% CI (1.11–7.03)] (2, 36). This could be attributed to the fact that, as part of PNC services, health professionals have been educating and advising mothers about complementary feeding practices.

In contrast, no statistically significant relationship was found between mothers’ educational status in Lalibela, Mekele, and Arisi Negele and household family size in Axum town and Lasta and the timely initiation of complementary feeding practice of children aged 6–23 months (19, 31, 33).

### Policy implications

In this study, failure to initiate complementary feeding on time was found to be a risk factor for acute malnutrition in children aged 6–59 months. This could be due to the fact that children above the age of 6 months require supplemental food to meet their nutritional demands, and initiating complementary feeding at a later stage can lead to undernutrition.

### Limitations of the study

One of the major limitations of the study was that the reported time to start complementary feeding was recorded without any means of verification. A cause-and-effect relationship could not be established because the study was cross-sectional in nature.

## Conclusion and recommendation

Finally, the prevalence of timely complementary feeding initiation was found to be lower than the WHO cutoff threshold for acceptable complementary feeding practices. As a result, it is critical to initiate immediate measures to promote adequate complementary feeding. This will have an impact on the health of newborns and young children. The prompt initiation of complementary feeding was greatly influenced by the mother's occupation, radio accessibility, ANC and PNC follow-ups, and birth location. In the future, it is crucial to take into account the following important activities to increase the percentage of people who start complementary feeding practices on time. The following recommendations are provided to the zonal health department, District Health Office, and appropriate health institutions on the basis of the current study findings.

• All health workers should be trained to focus on the timely implementation of complementary feeding practices, and efforts should be made to increase the utilization of maternal healthcare services.
• Maternal healthcare professionals should provide nutrition education with an emphasis on the timely initiation of complementary feeding by using mass media outlets, by creating and disseminating appropriate information, education, and communication (IEC) materials, and by using behavioral change communication (BCC) to raise awareness of appropriate feeding practices.
• Health professionals and health extension workers should focus their efforts on aggressively searching for pregnant mothers through the health development army network and providing proper counseling to them.
• Health extension workers should distribute IEC and BCC materials to households and counsel them.

## Data Availability

The original contributions presented in the study are included in the article/supplementary material, further inquiries can be directed to the corresponding author/s.

## Author’s note

AA has an MPH in Public Health Nutrition, SH has an MPH in Public Health Nutrition, and MT has an MSc in Infectious and Tropical Disease.
